# Nanomaterials and Devices for Harvesting Ambient Electromagnetic Waves

**DOI:** 10.3390/nano13030595

**Published:** 2023-02-02

**Authors:** Mircea Dragoman, Martino Aldrigo, Adrian Dinescu, Dan Vasilache, Sergiu Iordanescu, Daniela Dragoman

**Affiliations:** 1 National Institute for Research and Development in Microtechnologies, Erou Iancu Nicolae Street 126A, 077190 Voluntari, Ilfov, Romania; 2 Physics Faculty, University of Bucharest, 077125 Bucharest, Ilfov, Romania; 3 Academy of Romanian Scientists, Str. Ilfov, Nr. 3, 050044 Bucharest, Ilfov, Romania

**Keywords:** rectenna, 2D materials, energy harvesting

## Abstract

This manuscript presents an overview of the implications of nanomaterials in harvesting ambient electromagnetic waves. We show that the most advanced electromagnetic harvesting devices are based on oxides with a thickness of few nanometers, carbon nanotubes, graphene, and molybdenum disulfide thanks to their unique physical properties. These tiny objects can produce in the years to come a revolution in the harvesting of energy originating from the Sun, heat, or the Earth itself.

## 1. Introduction

In this review, we consider energy harvesting from ambient electromagnetic (EM) waves, since this method, together with energy harvesting from heat, are the most promising approaches for renewable energy production after photovoltaic DC power production via solar cells. Furthermore, solar cells are not referred to here, since there are already a large number of reviews dedicated to these devices, which are widespread in daily life. Although solar cells have been commercialized for some time, the research for more efficient harvesting solutions is growing every year, supported by the discovery of new materials, such as perovskites; among the reviews on the latter type of solar cells, see [1]. At the end of 2020, solar cells provided about 1 TW of electrical energy around the globe [2].

**Figure 1:**
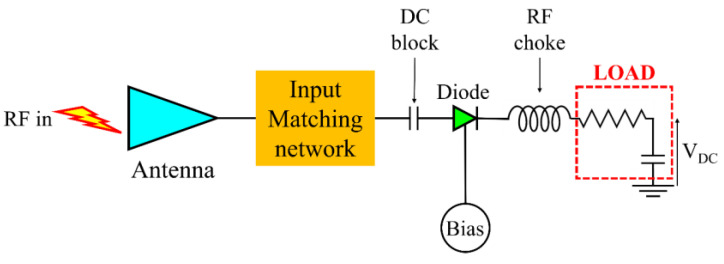
Schematic of the generic rectenna.

The radiofrequency (RF) energy harvesting (RF-EH) challenge originates from the unprecedented development of wireless high-frequency communications, such as 5G and 6G networks, able to generate an ambient EM energy available at any time and in a large band, spreading from the microwave spectrum up to sub-terahertz frequencies. This EM energy can be used on demand by equipment such as smartphones, tablets, laptops, and receivers at various frequencies, but a large part of it is not used and, hence, is lost. Harvesting the fraction of EM energy that is unexploited allows the self-powering of low-consumption electronic devices, e.g., those connected within Internet of things (IoT) architectures. The power supply mechanism associated with RF-EH techniques is based on a rectenna, i.e., an antenna loaded with an (un)biased diode [3,4,5]. Figure 1 shows the schematic of a generic rectenna, in which the antenna provides the RF input to a rectifying diode, whose DC output voltage *V_DC_* is the voltage drop on the load capacitor. A DC block capacitance and a RF choke inductance are (ideally) necessary at the input and output, respectively, of the diode to let only the DC component of the received RF signal pass. Furthermore, the diode should work in the unbiased state; however, in some circumstances (e.g., when the power at the input of the diode is too low to allow the device working in forward conduction), a small DC bias voltage could be necessary to force the rectifier to operate in its nonlinear region (this bias could be provided by EH sources other than the RF ones). Depending on the requested DC power, these rectennas can be arranged in arrays with various configurations involving one or more diodes. Many new materials are involved in the gathering of this ambient EM energy, such as two-dimensional (2D) materials (e.g., graphene and MoS_2_) [6,7], oxides [8], and nanoscale ferroelectrics (e.g., HfO_2_-based ferroelectrics) [9].

The heat is another source of ambient energy produced by cars, computers, houses, and the Earth, and this energy is basically lost in a large proportion. There are two main methods that are investigated here to produce DC power from heat. The first method is thermoelectric power generation (TEP), meaning the generation of a DC voltage difference Δ*V* due to a temperature difference Δ*T*, i.e., Δ*V* = *S* Δ*T*, where *S* is the Seebeck coefficient. The 2D materials are especially investigated for tiny TEP generators [10].

The second method is based on the pyroelectric effect, which entails the production of a time-variant current *i*(*t*) that depends on the time-dependent variation of the temperature *T*, as follows: *i*(*t*) = *α_p_ A* d*T*/d*t*, where *A* is the area of the device, and *α_p_* is the pyroelectric coefficient [11]. It was demonstrated that the pyroelectric effect is more efficient than TEP [12]. Nanoscale ferroelectrics such as HfO_2_-based ones are the most studied materials today for efficient pyroelectric generation [13,14].

Nanomaterials and devices suitable for heat harvesting do not explicitly constitute the subject of this paper, because we focus here on harvesting from EM fields, but they can be regarded of interest from the perspective of harvesting of infrared radiation.

## 2. Rectennas for Microwave, Millimeter-Wave, and Solar Cell-Like Harvesters

### 2.1. Rectennas Based on Schottky and MIM Diodes

Electromagnetic energy harvesting at microwaves and millimeter waves implies various antenna geometries integrated with one or more diodes for rectifying the incoming EM field at zero bias, devices termed as “rectennas”. The source of energy of microwave and millimeter-wave harvesters is the environmental EM energy produced by wireless communications, radars, and various emitting stations. 

Going to THz frequencies, the main energy source is the Sun, which generates a power density of 1000 W/m^2^ on Earth, at sea level, in good weather conditions. More than 50% of this energy is in the infrared (IR) spectrum, which is mainly lost by solar cells as they work with the highest efficiency in the visible spectrum. In particular, the 28 THz band represents a target for rectennas since, in this band, the IR is re-irradiated from the Earth. Such a harvester may be capable of functioning day and night in any weather conditions, in deep contrast to solar cells.

**Figure 2:**
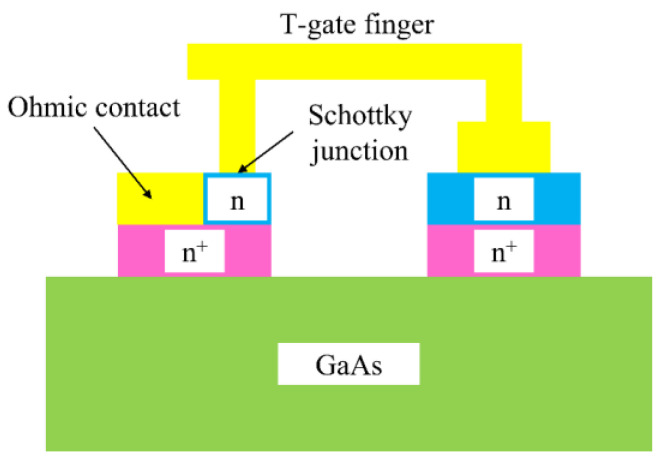
Schematic of a THz Schottky diode [19].

Indeed, rectennas are not only used in the microwave spectrum; in fact, they were designed to transform solar energy into DC, having a similar role to solar cells [15] (the history of rectennas can be found in [16]). The most common approach for rectennas in the microwave and millimeter-wave range is to integrate antennas with Schottky diodes; in this way, an integrated circuit fully compatible with the CMOS process can be achieved [17] up to 100 GHz [18], even reaching a few terahertz [19]. Figure 2 represents the schematic of a THz Schottky diode on a gallium arsenide (GaAs) substrate, using thin *n*^+^-doped and low *n*-doped epitaxial layers [19].

Moreover, there are new Schottky diodes based on chrome–silicon (Cr–Si) and cobalt–silicon (Co–Si), which are able to detect IR radiation [20]. New materials and new substrates are searched for constantly in EM harvesting. When very high levels of EM power are harvested, Schottky diodes based on diamonds are used [21], while, at THz frequencies, graphene diodes have been proposed [22]. In the RF range up to 1–2 GHz, various substrates have been exploited, such as paper [23,24], textiles [25], metamaterials [26], or flexible substrates such as polyimide [27]. An updated account of rectennas and their applications can be found in [4]. There is a wide range of solutions for rectennas depending on the targeted bandwidth. 

**Figure 3:**
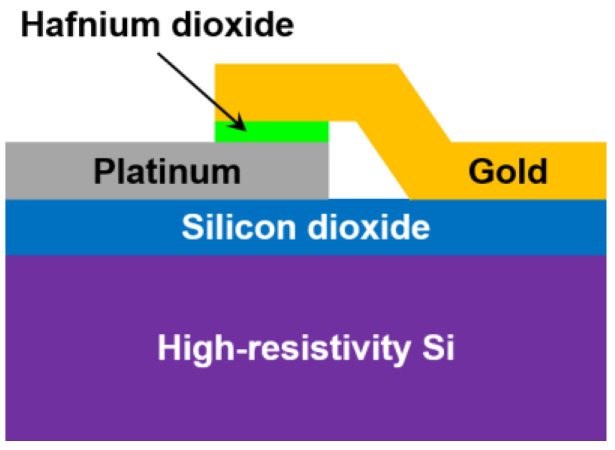
Cross-section of an HfO_2_-based MIM diode. Reproduced with permission from [29].

Metal–insulator–metal (MIM) diodes are also used for rectennas, since they are the only electronic devices able to work up to visible and infrared wavelengths [28] because the corresponding tunneling time is on the order of a few femtoseconds. The MIM depicted in Figure 3 is a very simple electronic device at the conceptual level, but it is also quite difficult to fabricate if one wants to achieve a high yield. The reasons are manyfold: (i) the insulator must have a thickness of a few atoms (which could be damaged during the patterning process, e.g., by dry or wet etching techniques, depending on the material); (ii) the contact area must be a few μm^2^ down to a few tens of nm^2^ (depending on the cutoff frequency); (iii) there is a need to configure the top electrode as a bridge, which is a very delicate task, as it could generate undesired short-circuits.

The above MIM configuration is a device where the insulator is a very thin (6 nm) dielectric hafnium oxide (HfO_2_) layer and where the two metals for the bottom and top electrodes are platinum (Pt) and gold (Au), respectively. HfO_2_ is grown using the atomic layer deposition (ALD) technique, which guarantees a very low surface roughness of 0.1–0.2 nm; this is a fundamental prerequisite for any MIM diode, which ensures that the tunneling phenomenon is not suppressed by a too high roughness. The high-resistivity silicon (HRSi) substrate is mandatory for the devices to work at high frequencies (a low-resistivity silicon wafer would totally hinder the microwave and millimeter-wave propagation).

**Figure 4:**
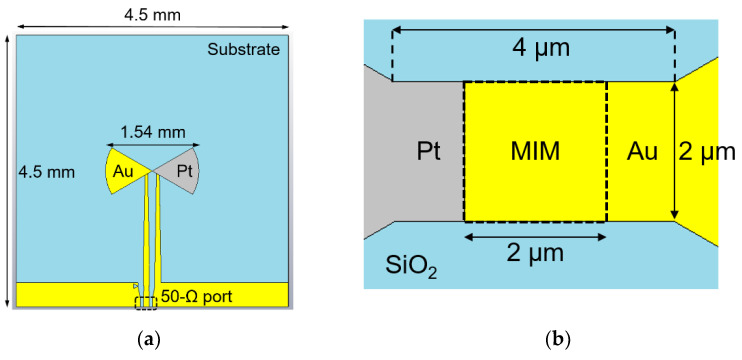
EM layout of the 60 GHz HfO_2_-based rectenna: (**a**) top-view; (**b**) MIM area. Reproduced with permission from [29].

In Figure 4, we illustrate a rectenna fabricated at the wafer level for the 60 GHz band [29]. The rectenna consists of a bowtie antenna integrated with the Au/HfO_2_/Pt MIM diode depicted in Figure 3. The top and bottom electrodes of the HfO_2_ MIM diode are the bowtie antenna arms. One rectenna has a surface of 20.25 mm^2^ and has a length of 1.54 mm. The substrate is an HRSi wafer having a thickness of 525 μm, over which a 300 nm thick film of silicon dioxide (SiO_2_) is thermally grown.

**Figure 5:**
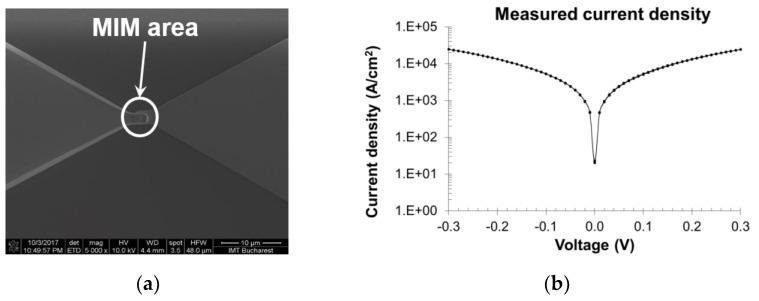
(**a**) SEM image of the MIM area of the 60 GHz rectenna; (**b**) measured current density–voltage dependence of the MIM diode in the range [−0.3, 0.3] V. Reproduced with permission from [29].

The rectenna output is designed in the coplanar waveguide (CPW) technology for on-wafer characterization, and its fabrication is detailed in [29]. In Figure 5a, a scanning electron microscope (SEM) image of the bowtie antenna is displayed, whereas Figure 5b reports the measured current density–voltage dependence of the MIM diode.

A typical parameter for a rectifying diode is the differential resistance *R_D_*, defined as follows:

*R_D_* = 1/(*∂I*/*∂V*), (1)

where *I* is the current, and *V* is the voltage. *R_D_* is important because it affects the matching to antenna’s input impedance and, therefore, the overall RF-to-DC conversion efficiency of the harvester.

In the present case study, we have *R_D_*_0_ = *R_D_*(*V* = 0) = 405 Ω, whereas, at ±100 mV, the differential resistance is *R_D_* = 300 Ω, and, at ±300 mV, we have *R_D_* = 92 Ω. Since the bowtie input impedance is around 300 Ω, we can state that we have a good coupling to the antenna’s input impedance even at 0 V; moreover, if the diode is slightly biased, the coupling improves to ±100 mV.

There are two important figures of merit for MIM diode characterization, the nonlinearity *χ* and curvature coefficient (or sensitivity) *γ*, which are defined as follows [29]:

*χ* = (*∂I*/*∂V*)/(*I*/*V*), (2)

*γ* = (*∂*^2^*I*/*∂V*^2^)/(*∂I*/*∂V*). (3)

**Figure 6:**
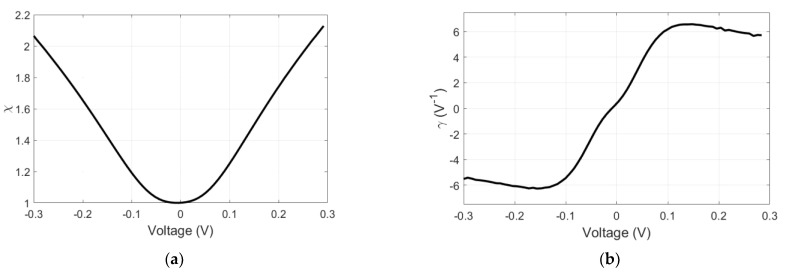
(**a**) Nonlinearity *χ* and (**b**) sensitivity *γ* of the measured HfO_2_-based MIM diode. Reproduced with permission from [29].

The two figures of merit are represented in Figure 6.

**Figure 7:**
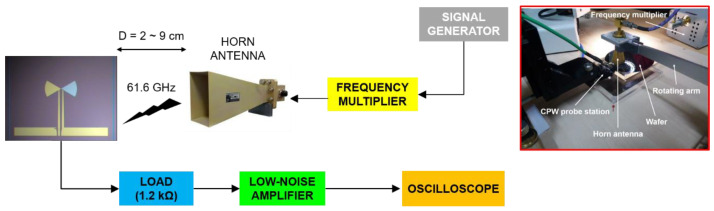
(**Left**) Schematic and (**right**) picture of the measurement system used to characterize the 60 GHz rectennas. Reproduced with permission from [29].

**Figure 8:**
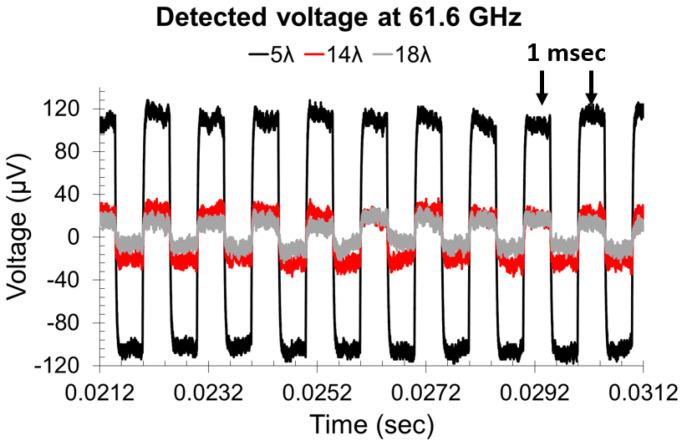
Measured detected voltage from the 61.6 GHz transmitter at various distances in terms of number of wavelengths. Reproduced with permission from [29].

The experimental setup used for measuring the above rectennas is displayed in Figure 7. The input signal is an amplitude-modulated (AM) sinusoidal at 61.6 GHz with a modulation rate of 1 kHz (Figure 8). With such rectennas, we can harvest up to 250 μV at a distance of fivefold the free-space wavelength λ_0_ (at the fundamental frequency) from the millimeter-wave transmitter, with a responsivity of 5.4 V/W at an incident power of −20 dBm.

This is one of many examples of rectennas using MIM diodes, with a recent review available in [8]. We note that rectennas integrated with MIM diodes can also work at 1–3 THz and at 28 THz. Various thin oxides are used to attain this goal. The most known are NiO, Al_2_O_3_, and HfO_2_. Their thickness must be in the range 2–6 nm to ensure the occurrence of the tunneling effect, with the actual thickness depending also on the roughness of the metal that is used (a low roughness allows the deposition of thinner insulator layers). IR rectennas based on oxides are being further developed on the basis of more efficient MIM structures, such as MI^n^M (where “I^n^” indicates an n-layer insulator), i.e., relying upon oxide heterostructures to increase the nonlinearity of the diode and its cutoff frequency [30].

### 2.2. Rectennas Based on 2D Materials

Two-dimensional materials are considered promising candidates for RF harvesting. Graphene monolayers have been (and still are) the object of extensive research thanks to their high carrier mobility and the control of the carrier density by means of gate voltages, but mostly for the ballistic transport at room temperature over a very large distance in comparison to any other material. Some physical properties of graphene are displayed in Table 1, whereas Table 2 lists the most used growth methods.

**Figure 9:**
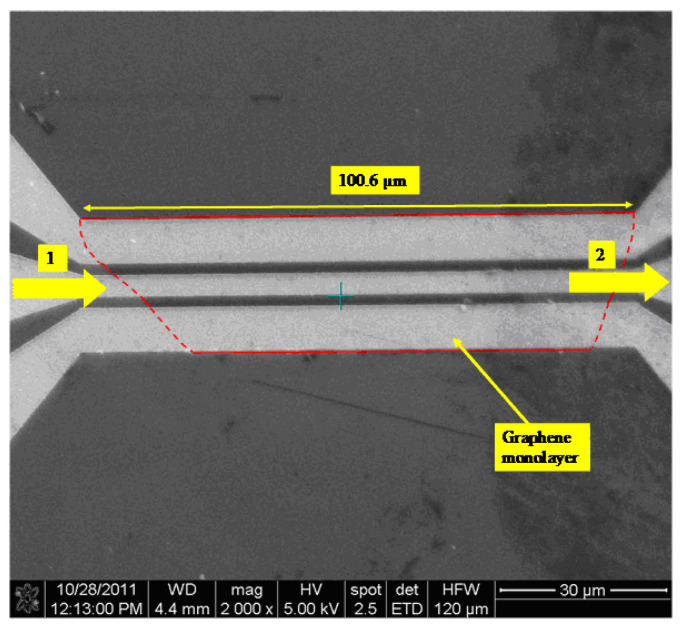
SEM image of the CPW-on-graphene microwave harvester. Reproduced with permission from [32].

**Figure 10:**
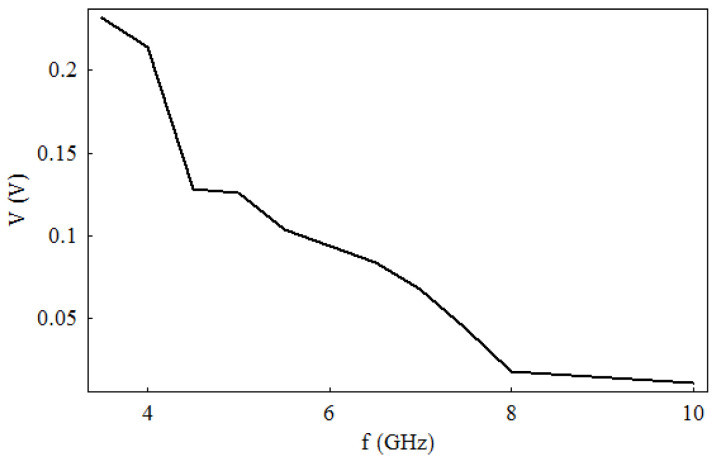
DC voltage at the output of the CPW-on-graphene microwave harvester. Reproduced with permission from [32].

If we deposit on a graphene monolayer three metallic electrodes (made of chrome/gold (Cr/Au) with a thickness of 0.2–1 μm) in a CPW configuration, then this simple device acts as a harvester for microwave signals [32]. Figure 9 presents such a CPW-on-graphene microwave harvester, which is able to detect a DC voltage in the range 0.005–0.2 V (without any applied bias) when excited with AM microwave signals in the 3–10 GHz band with a power of 0 dBm, where the modulation signal constitutes audio pulses with frequencies higher than 1 kHz (Figure 10).

**Figure 11:**
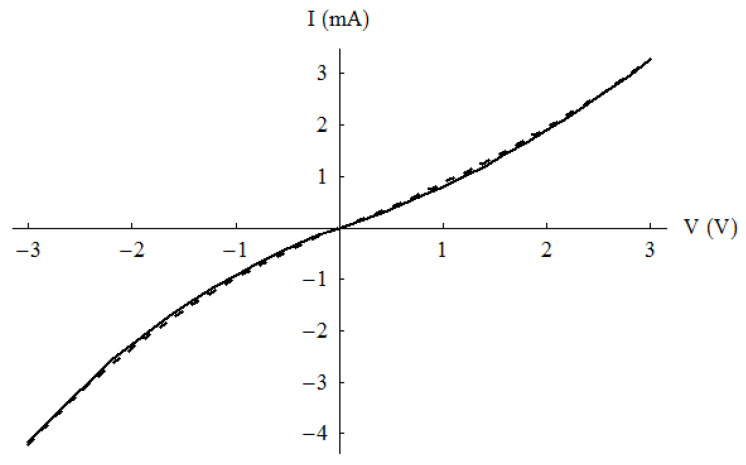
Nonlinear current–voltage dependence of the CPW-on-graphene microwave harvester. Solid lines: measured data; dotted lines: analytical results from Equation (4). Reproduced with permission from [32].

The explanation of this result is the nonlinear current–voltage dependence (Figure 11) of the CPW-on-graphene device, which is described by the following formula:

*I = I*_0_ [exp (*V*/*V*_0_) − 1], (4)

where *I*_0_ and *V*_0_ have the values of 3.65 mA and 4.68 V, respectively, for positive voltage values, and −2.6 mA and −3.12 V, respectively, for negative voltages.

**Figure 12:**
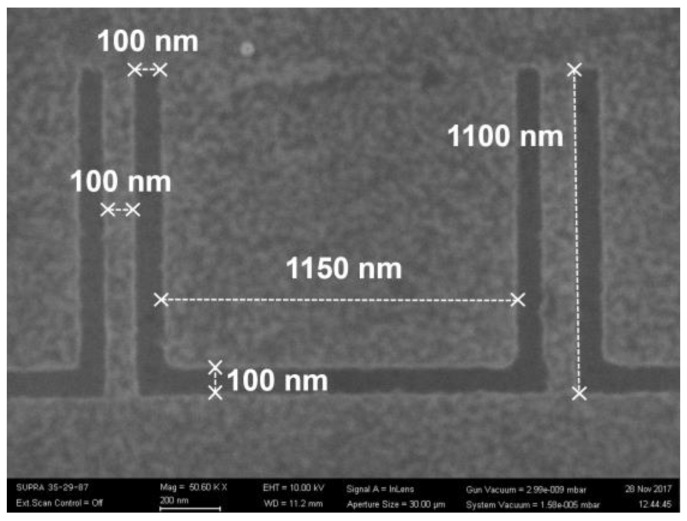
SEM image of a detail of two adjacent channels of the fabricated graphene-based SSD. Reproduced with permission from [41].

A new type of rectenna based on so-called self-switching diodes (SSDs) [33], which are planar semiconductor/2D material structures, is expected to work even at THz frequencies thanks to their operating principle [34], based on nonlinear current flow through nanometer-sized parallel channels. The flow control is assured by DC field effects. An SSD is similar to a double side-gated field-effect transistor, which becomes a diode by simultaneously shortcutting the drain and the gates [35]. The SSD is in fact a geometrical diode; there are no junctions, and no doping is necessary. SSD detectors have been experimentally demonstrated using various materials, such as graphene for microwave and millimeter waves [36,37]: InP/InGaAs/InP, AlGaN/GaN, and InAs [38,39,40]. In [41], we reported the fabrication at the wafer level of harvesters based on SSDs in the Ka band (28 GHz) integrated with a four-element patch antenna array. The substrate was a 4 inch HRSi/SiO_2_/graphene monolayer wafer, with the HRSi having a thickness of 525 μm and a resistivity of 10,000 Ω·cm, while the SiO_2_ layer had a thickness of 300 nm. The graphene was transferred on the HRSi/SiO_2_ wafer by Graphenea (San Sebastian, Spain). The graphene-based SSD is presented in Figure 12.

The modeling of the graphene-based SSD was described in [37]. The drain current (*I_d_*)–drain voltage (*V_d_*) dependence is given by

**Figure 13:**
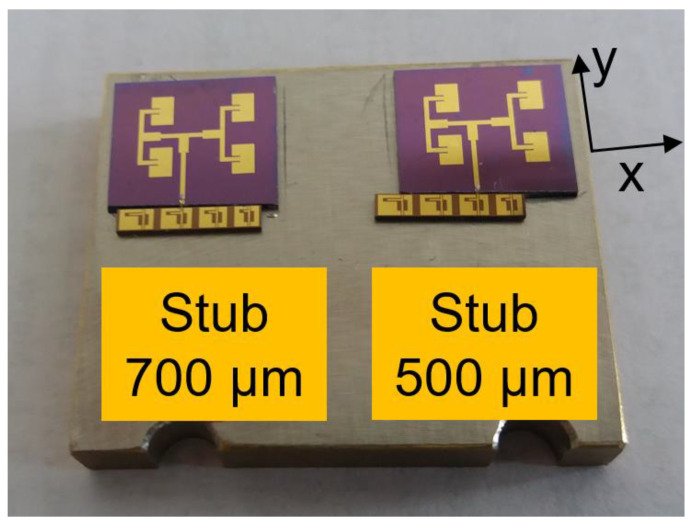
Fabricated 28 GHz rectennas based on graphene-based SSDs. Reproduced with permission from [41].

*I_d_ ≈ N μ C_s_ w / l |V_d_ – V_D_| V_d_,* (5)

where *N* is the number of channels, *μ* is the channel mobility, *w* is the distance between adjacent channels, *ℓ* is the length of the channel, and *V_D_* is the Dirac voltage. After an optimization procedure focused on the maximization of the current at low voltage values, we found *N* = 12, *w* = 100 nm, and *ℓ* = 1.1 μm. Thus, the graphene-based SSD is formed by 12 channels in parallel; in this way, the total current is the sum of currents flowing in all channels (which also entails a decrease of the total resistance). The experimental characterization of the graphene-based SSDs in DC and at microwaves was performed using CPW lines with open stubs of different lengths (namely, 300, 500, 700, and 900 μm) for matching to 50 Ω. The fabrication of the rectenna was described in [41], and the fabricated devices are presented in Figure 13.

**Figure 14:**
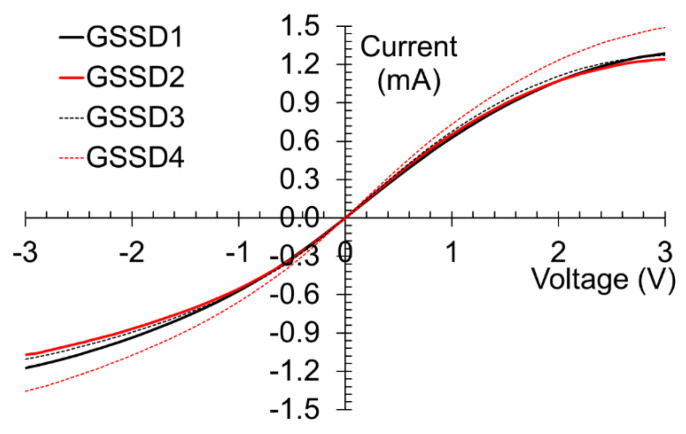
Current–voltage dependence of several graphene-based SSDs. Reproduced with permission [41].

The current–voltage dependence is displayed in Figure 14 for four different diodes, denoted as GSSD1 to GSSD4. The DC current has a maximum value of ±1 mA at ±3 V, one of the best results obtained so far for SSDs thanks to the optimization strategy described above.

The array consists of four patch antennas (in a 2 × 2 configuration) made of a 500 nm thick Au layer. The length and width of each patch antenna are *L* = 1.5 mm (*x*-direction) and *W* = 2.206 mm (*y*-direction), respectively. The spacing between antennas is about 0.51*λ*_0_ along the *x*-direction and 0.37*λ*_0_ along the *y*-direction, where *λ*_0_ ≈ 10.71 mm corresponds to the free-space wavelength at 28 GHz.

**Figure 15:**
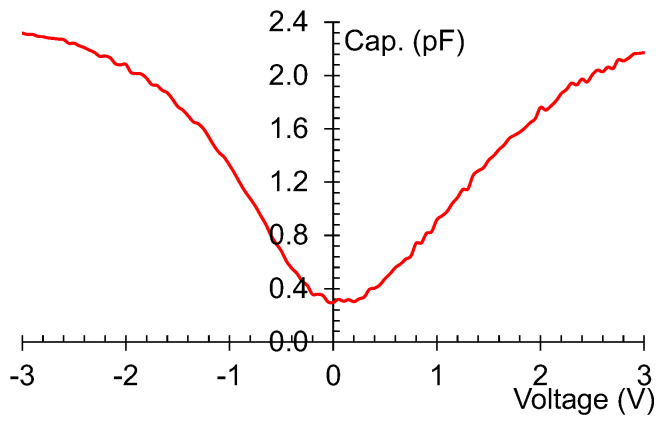
Capacitance-voltage dependence of the graphene-based SSDs. Reproduced with permission from [41].

In Figure 15, the capacitance–voltage (C–V) dependence is depicted at 1 MHz. We observe that the ratio between the maximum and minimum capacitance is 6, meaning that the graphene-based SSD works as a varactor. More details about the differential resistance *R_D_* = 1/(*∂I_d_*/*∂V_d_*) and intrinsic curvature coefficient *γ_i_* = (*∂*^2^*I_d_*/*∂V_d_*^2^)/(*∂I_d_*/*∂V_d_*) can be found in [41].

The RF characterization was performed using an Agilent analogue signal generator, connected to a horn antenna (with a gain of 24 dBi) and a rectenna integrated with a graphene-based SSD located in the far-field region of the horn antenna. The horn antenna was excited by a 28 GHz sinusoidal carrier modulated by a square AM signal of frequency *f_AM_* = 1 kHz. The effective RF input power delivered to the rectenna was in the range 13–470 μW. In this way, a maximum responsivity of 96.16 V/W at 28 GHz for an applied DC drain voltage bias *V_d_* = −1.35 V was calculated.

**Figure 16:**
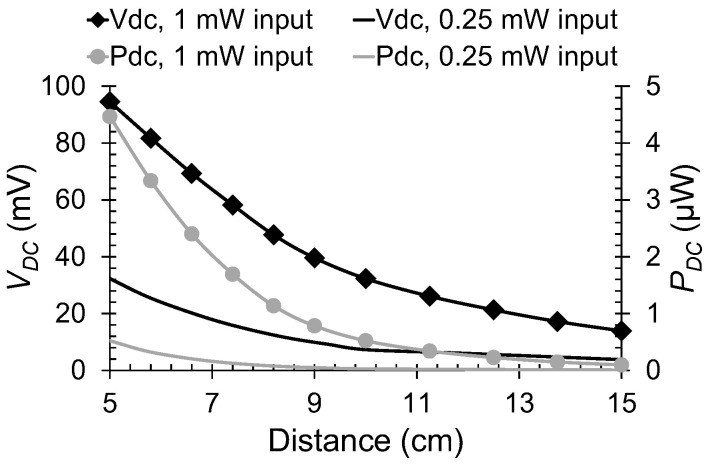
DC voltage (left axis, black lines) and power (right axis, gray lines) for two values of the transmitted power. Reproduced with permission from [41].

In Figure 16, we show the DC voltage *V_DC_* and power *P_DC_* of the rectenna dependence on the distance between the horn antenna and the rectenna, for *V_d_* = −0.66 V and for two values of the power at transmitter’s input (1 mW and 0.25 mW). It can be observed that the maximum values are *V_DC_* = 94.5 mV and *P_DC_* = 4.47 μW at 5 cm distance. The NEP (noise equivalent power) is just 692 pW∙Hz^–0.5^ for an RF input power of about 500 μW.

**Figure 17:**
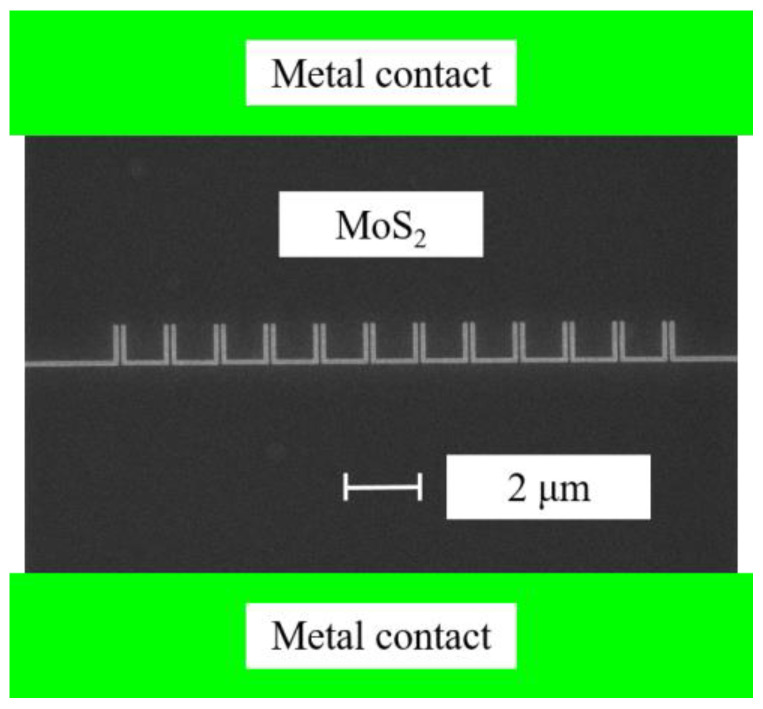
SEM image of the fabricated MoS_2_-based SSD detector [43].

Molybdenum disulfide (MoS_2_) is another 2D material candidate for rectennas. MoS_2_ (both monolayer and multilayer) is a semiconductor [42], while graphene is a semimetal. Today, high-frequency applications of MoS_2_ are rather poor due to difficulties in the growth of MoS_2_ at the wafer level and due to its low mobility, knowing that a high value of carrier mobility is a prerequisite for any semiconductor for microwave/millimeter-wave devices. A short overview of MoS_2_ for high-frequency applications can be found in [43] and the references therein. The state of the art for MoS_2_ in rectennas means the realization of zero-bias detectors, which can be further integrated with antennas to become rectennas, as explained before. In this respect, we grew a MoS_2_ thin film (nominal thickness of 10 monolayers) on a 4 inch Al_2_O_3_/HRSi wafer by chemical vapor deposition (CVD). The obtained zero-bias detector was a self-switching diode able to operate in the most used high-frequency band, i.e., in the range 0.9–10 GHz (Figure 17), where almost all wireless communications and radars are working.

**Figure 18:**
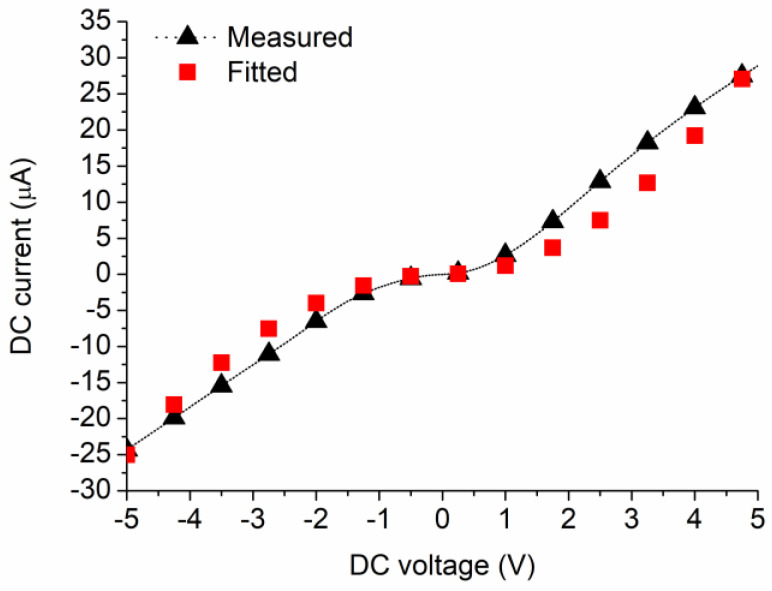
Current–voltage dependence of the MoS_2_-based SSD. Dotted line with black triangles: experimental results; red squares: fitted curve [43].

The MoS_2_-based SSD has a current dependence on voltage as *I* ⅙∝ *V*^2^, i.e., it works as a square law detector, where *V* is the applied voltage. The current–voltage dependence of the MoS_2_-based SSD is presented in Figure 18 together with a fitted curve of the type ±*αV*^2^. The excellent fit confirms that the diode works as a detector.

**Figure 19:**
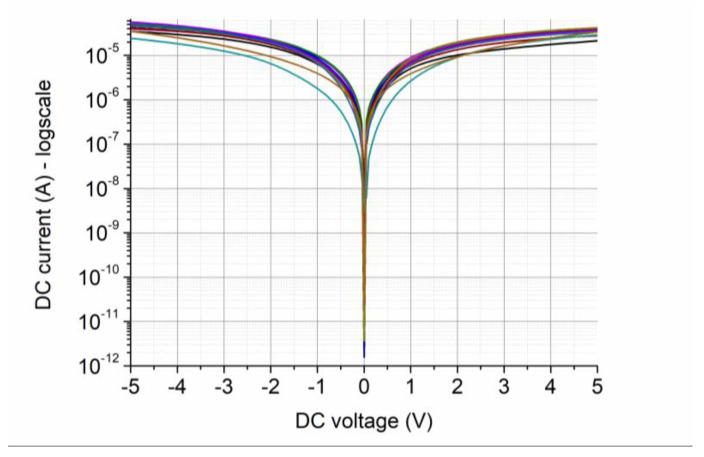
Current–voltage dependences of 10 distinct MoS_2_-based SSDs [43].

By representing the current in logarithmic scale in the current–voltage dependences of 10 distinct diodes (Figure 19), it can be seen that the on/off ratio is 10^6^.

**Figure 20:**
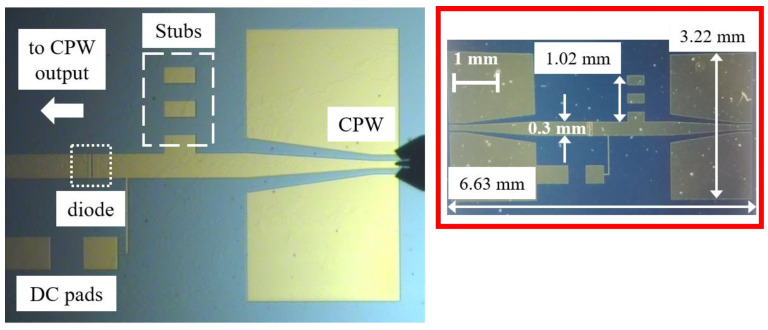
Picture of the zero-bias MoS_2_-based detector [43].

The complete circuit embedding the MoS_2_-based detector in CPW technology contains CPWs for on-wafer input excitation and output measurements, and it comprises tunable open stubs for matching to the SSD’s impedance and, hence, maximizing the power transfer (Figure 20).

**Figure 21:**
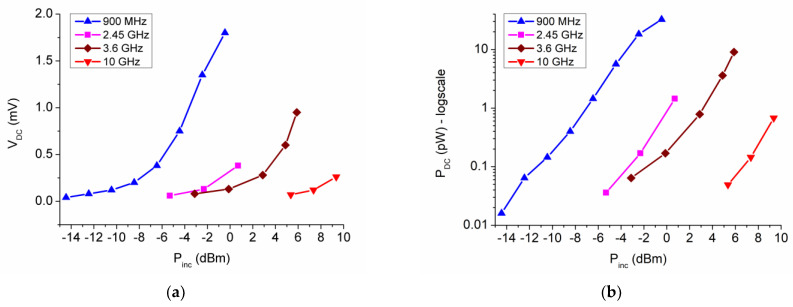
Performance of the MoS_2_-based SSD zero-power detector: (**a**) DC output voltage; (**b**) DC output power [43].

The zero-power detector was experimentally tested by varying the incident power between −15 dBm and +10 dBm for a couple of frequencies in the range 900 MHz–10 GHz; the results are shown in Figure 21. We observe that the increase of DC voltage is possible only by increasing the microwave power. The DC voltage generated only by microwave signals is sufficient to bias low-power electronic devices such as microprocessors [44]. 

Furthermore, a zero-power detector using a double-gate MoS_2_ FET was fabricated and tested at the wafer scale, with the substrate being an 8 μm-thick polyimide film on Si [45]. This detector exploits the nonlinear behavior of FETs beyond their cutoff frequency. The FETs are based on a MoS_2_ monolayer channel and show a responsivity of 45 V/W at 18 GHz, while, for a multilayer MoS_2_ channel, the corresponding parameter is 104 V/W at 16 GHz. To attain such good performances, the MoS_2_ channel is sandwiched between h-BN layers to decrease the interface effects. 

**Figure 22:**
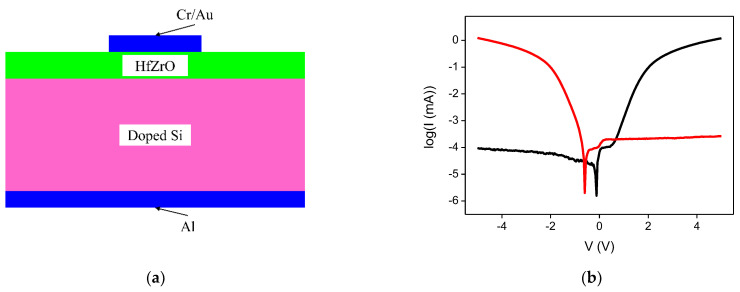
(**a**) Cross-section of the HfZrO-based FTJ; (**b**) current–voltage dependence [9].

Lastly, nanoscale ferroelectrics can also be used for harvesting RF energy. We are witnessing an extraordinary development of such materials and of their applications in various domains thanks to the discovery of new classes of nanoscale ferroelectrics with unexpected properties, such as HfO_2_-based ferroelectrics with a thickness in the range 1–6 nm, or 2D ferroelectrics extensively studied for new devices, e.g., memristors and memtransistors, negative capacitance transistors, and many other RF devices [46]. Among the latter, we can mention the ferroelectric tunneling junctions (FTJs) [47], which are MIM diodes where the insulator is a ferroelectric with a thickness of just a few nanometers. In FTJs, the current is switched on and off by the applied DC voltage, whose polarity switches the orientation of ferroelectric domains. In our case, we used poling voltage values of ±10 V to switch the ferroelectric domains of a zirconium-doped hafnium oxide ferroelectric (referred to as “HfZrO”) thin film-based FTJ, thus inducing a pronounced rectification of the current–voltage dependence of the FTJ, with an on/off ratio of 10^4^. The HfZrO-based FTJ is shown in Figure 22a (in which the top and bottom metallic contacts are Cr/Au and aluminum (Al), respectively), whereas the current–voltage dependence is shown in Figure 22b. Using the FTJ as a diode coupled to an antenna array to create a rectenna, the current values are on the order of milliamperes, and we can harvest EM energy at 26 GHz (a bandwidth assigned to IoT), obtaining a responsivity of 63 V/W and an NEP of 4 nW∙Hz^–0.5^ [9].

**Figure 23:**
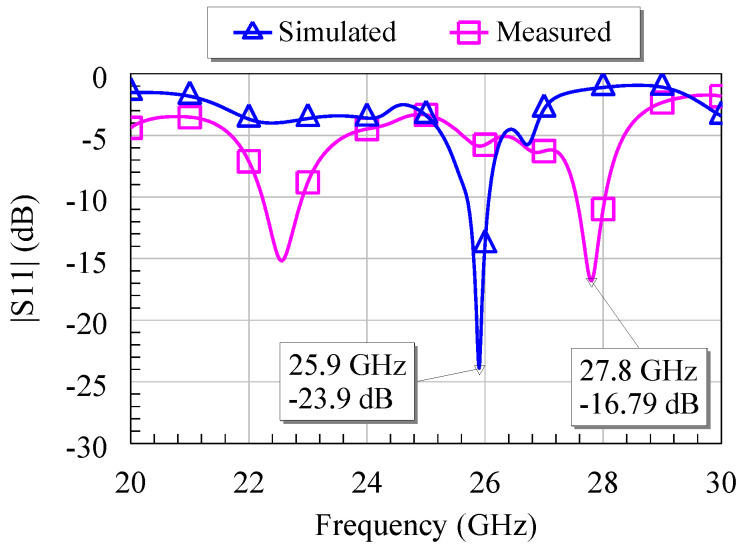
Reflection coefficient |S_11_| of the fabricated antenna array [9].

**Figure 24:**
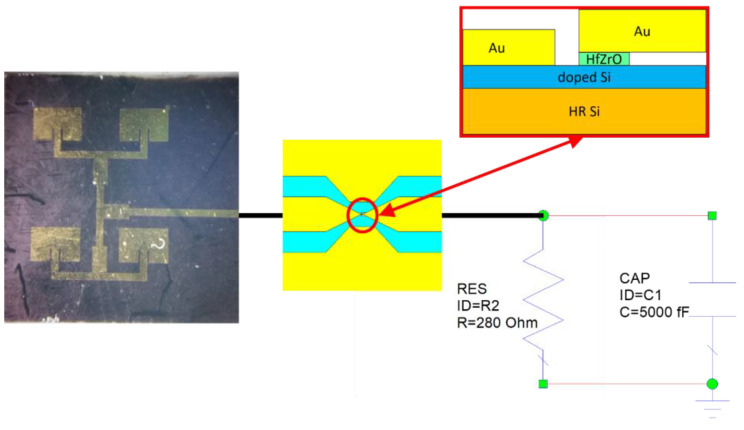
Fabricated rectenna consisting of four patch antennas and an HfZrO-based FTJ [9].

The antenna array was fabricated on a SiO_2_ layer (thickness of 300 nm) grown on a 525 μm thick HRSi wafer. The metallization of antennas consists of a 500 nm thick Au layer. The width and length of each patch antenna are *W* = 2.22 mm and *L* = 1.46 mm, respectively, and the spacing between antennas is about *λ*_0_/2 in the (*x*,*y*) directions, with *λ*_0_ ≈ 11.54 mm being the free-space wavelength at 26 GHz. The antenna array input impedance is 50 Ω. The reflection coefficient |S_11_| of the array is presented in Figure 23. Since |S_11_| = −15 dB at 22.2 GHz and |S_11_| = −16.79 dB at 27.8 GHz, the operating frequency was chosen to be 27.8 GHz. The fabricated rectenna is presented in Figure 24.

Lastly, we must point out that there are many reviews dedicated to harvesting microwaves and millimeter waves based on antennas integrated with semiconductor rectifiers of various types. Here, we focus on rectifiers based on nanomaterials, while the antennas are the same as in other classical solutions reported in the literature [48,49,50].

### 2.3. Rectennas for THz Frequencies Using Nanomaterials

In Table 3, we present the main sources for EM harvesting [51]. We can observe from this table that, to reach the most powerful source of energy located at very high frequencies, i.e., tens or even hundreds of THz, we need to downscale the rectenna’s dimensions. However, this downscaling is not easy to achieve due to several physical and technological obstacles: (i) the antenna’s dimensions become very small, and their reproducibility (as well as of the rectifiers with a time response in the order of femtoseconds) at the nanoscale is a real problem; (ii) at infrared frequencies, the EM field penetrates noble metals, creating very high EM losses; yet a replacement of noble metals with others, showing lower losses, is impracticable since Cu and Al, for instance, are quickly oxidized.

Moreover, the impedance mismatch between antennas or any type of EM collector (plasmonic devices, resonators, etc.) and the rectifier becomes very critical, which causes the conversion efficiency to decrease from 80% in the case of microwave rectennas [52] to 0.01% in the case of the best infrared CMOS-compatible rectennas [53]. We stress here that the power conversion efficiency is the ratio between the detected DC power and the EM source power.

There are series of reviews and books about antennas for THz, infrared, and optical frequencies; thus, we do not focus here on such a developed subject [54,55,56,57]. We concentrate instead on the electronic devices able to rectify such ultrahigh frequencies.

MIM diodes have been used for many years mainly for THz and infrared detection, e.g., using a 1 nm thick NiO layer as insulator [58]. Many other thin oxides with a thickness in the range 1–3 nm have also been used over the years with satisfactory results [8]. Beyond these results, there are several new solutions based on advanced knowledge of nanomaterials. 

A rectenna operating in the visible and infrared wavelength range needs to work at frequencies on the order of PHz (i.e., 10^15^ Hz); hence, the diodes must operate at speeds corresponding to few femtoseconds. The cutoff frequency of a rectenna integrating an antenna and a MIM diode is given by

**Figure 25:**
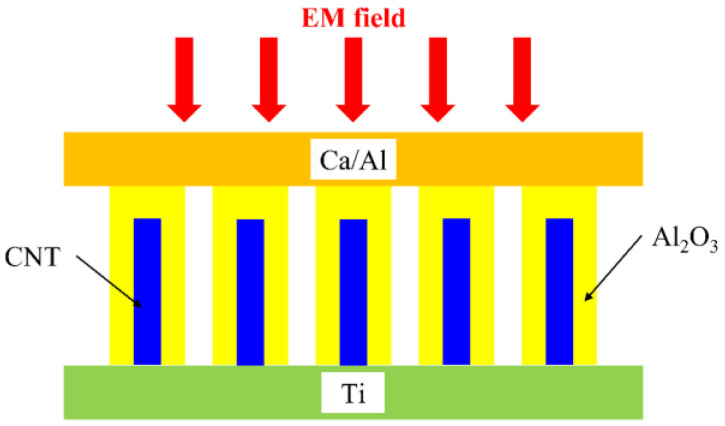
CNT-based optical rectenna.

*f_c_* = 1/(2*πR_A_ C_D_*), (6)

where *R_A_* and *C_D_* are the antenna resistance and diode capacitance, respectively. From this formula, we can see that attaining 1000 THz requires a capacitance of a few attofarad. To solve such difficulties, an array of carbon nanotubes (CNTs) antennas embedded into an Al_2_O_3_ dielectric layer and sandwiched between two metallic electrodes (Figure 25) was implemented, which constitutes the first optical rectenna [59]. The top electrode is made of calcium/aluminum (Ca/Al) to make it transparent, whereas the bottom electrode is made of titanium (Ti). However, metallic Ca is unstable in air; thus, the reliability of the entire optical rectenna is affected. The CNTs have a diameter of 10 nm and a height of 10 μm, and they are covered with 8 nm of Al_2_O_3_. The photo-response at 532 nm is 5 A/W, while, at 1064 nm, it becomes 1.4 A/W. The open DC voltage collected from a sun-like source is 0.52 mV, with an overall efficiency of just 10^–5^%.

**Figure 26:**
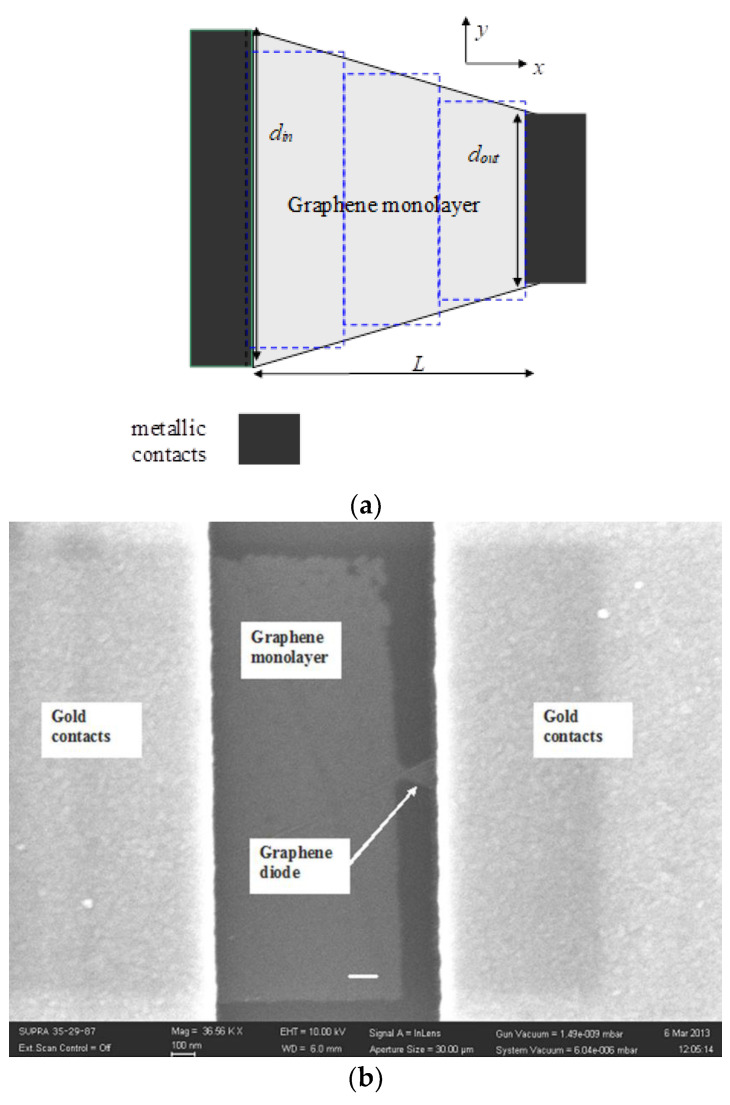
(**a**) Schematic and (**b**) SEM image of the graphene ballistic diode. Reproduced with permission from [60].

Another solution to attain THz frequencies is to use rectennas based on geometric diodes. Geometrical diodes induce rectification only due to their shape. This is possible in graphene due to its ballistic transport characteristics [60,61]. An example of a funnel geometric diode is presented in Figure 26. The current–voltage dependence of the graphene diode is computed using the Landauer formula [62], and it is found by dividing the total area of the diode in a certain number of regions, solving the Dirac equation in each region, and imposing the continuity conditions at each interface for the spinorial solutions of the Dirac equation. It is found that the diode has a certain region of width πℏ*v_F_*/*d_out_*, where *v_F_* is the Fermi velocity in graphene, where the current is nearly zero. Hence, the diode is rectifying in this region, and its current–voltage characteristic is strongly dependent on the Fermi energy, which can be tuned by a gate voltage. We fabricated the geometric diode on a graphene monolayer wafer deposited on a 4 inch wafer purchased from Graphene Supermarket (Ronkonkoma, NY, USA). The graphene monolayer was grown by CVD and transferred on 285 nm of SiO_2_ grown on *p*-doped Si. PMMA was spin-coated over the graphene wafer, the geometrical diode was patterned with an e-beam tool (Raith e-Line), and RIE equipment was deployed for cutting the graphene into trapezoidal shapes. Then, PMMA coating and e-beam lithography were again employed to obtain the metallic contact patterning. The metal deposition was performed in a highly directional e-gun evaporation chamber (Temescal), and the liftoff process was performed in acetone.

**Figure 27:**
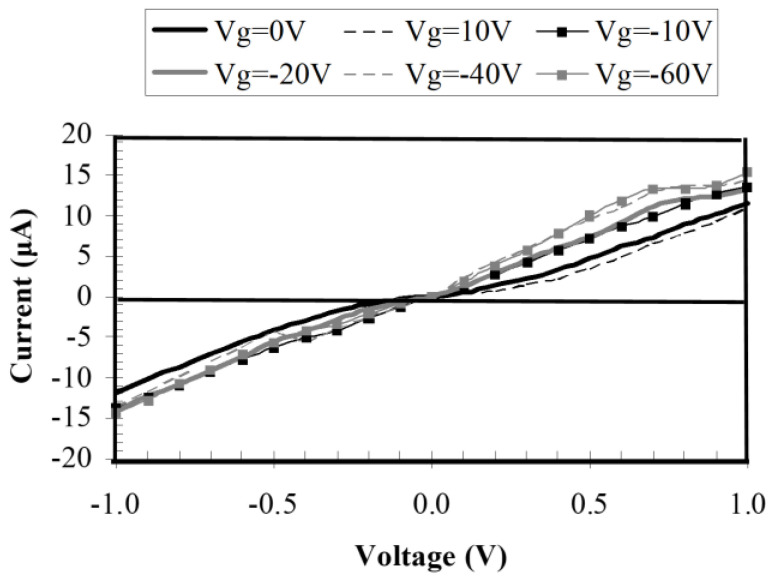
Current–voltage dependence of the ballistic graphene funnel diode. Reproduced with permission from [60].

**Figure 28:**
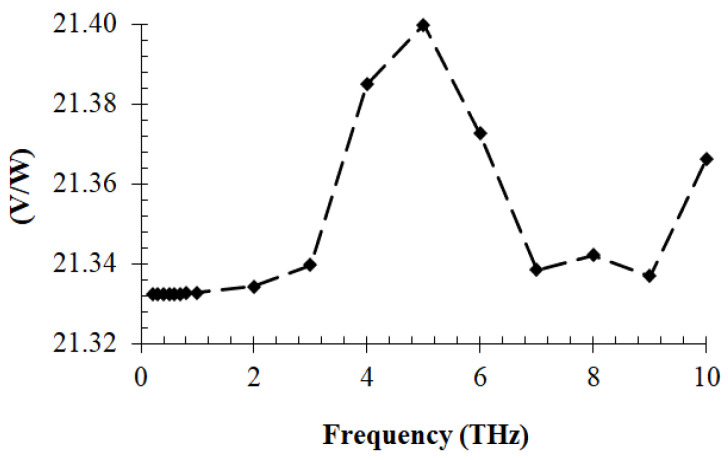
THz responsivity of the graphene geometric diode. Reproduced with permission from [60].

The current–voltage dependence of the geometric graphene diode is depicted in Figure 27 at various gate voltage values. Connecting this diode with THz dipole antennas on graphene, a good responsivity was obtained (Figure 28). The matching problem of the diode to the antenna can be solved by considering that the diode has an input resistance around 64 kΩ, but the dipole exhibits a large reactive part, on the order of 5–10 kΩ. Thus, to match the dipole antenna to the graphene diode around 8–10 THz, we used very small transmission lines for the two parallel inductances of 0.5 nH connected to the antenna, ending with a capacitor of 0.5 fF, which in turn was connected in parallel with the 64 kΩ diode. Thus, at 8.1 THz and 8.8 THz, we obtained a very good reflection coefficient, of −13.3 dB (VSWR = 1.53). The responsivity is displayed in Figure 28.

In [57], a similar graphene geometric diode was connected to a bowtie antenna since its RC constant was attained just a few femtoseconds thanks to the ballistic transport regime in graphene. At 28 THz and at zero bias, the current responsivity was 0.02 A/W using a CO_2_ laser as a source.

**Figure 29:**
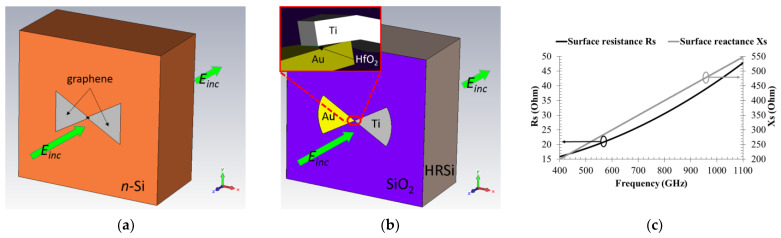
EM layout of the simulated graphene self-rectifying rectennas: (**a**) graphene bowtie antenna on *n*-doped Si; (**b**) equivalent Au/Ti bow-tie antenna on SiO_2_/HRSi with a thin layer of HfO_2_ in the gap mimicking a MIM diode; (**c**) computed values of graphene’s complex surface impedance in the 400–1100 GHz frequency range, where the solid black curve is the surface resistance *R_S_*, and the solid gray curve is the surface reactance *X_S_*. Reproduced with permission from [22].

Thus, the main obstacle against the high efficiency of rectennas working in the THz frequency range is the impedance mismatch between antenna and diode. Consequently, the conversion efficiency is low, while, in the case of microwaves, the same parameter can reach values beyond 80%. A solution to tackle this large discrepancy is to implement an antenna able to rectify the EM radiation [22]. This is possible using antennas made of graphene by transferring them onto an *n*-doped Si, GaAs, 4H-SiC, or GaN substrate, where a Schottky barrier at the graphene–semiconductor interface can form [63,64,65]. In this way, a graphene antenna acts as a receiver of EM energy and as a rectifier at the same time, without any additional diode. We obtained in [22] a maximum conversion efficiency of 58.43% at 897 GHz for the graphene rectenna on *n*-doped GaAs. We also compared our results with an MIM diode. We supposed that the one-atom-thick CVD graphene layer was transferred onto a 200 μm thick layer of *n*-type Si or *n*-type GaAs. The *n*-Si doped substrate has a doping of (2–6 × 10^15^ cm^−3^), relative permittivity of 11.9, and electrical conductivity of 33.333 S/m. The *n*-GaAs substrate is doped with Si (3–6 × 10^16^ cm^−3^) and has a permittivity of 12.94 and an electrical conductivity of 2000 S/m. The antenna is a graphene monolayer with a bowtie shape having the length 185 μm, with an aperture of 60° and a central gap region 7.82 μm long and 3.84 μm wide. Figure 29a shows the graphene bowtie antenna on *n*-Si, while Figure 29b presents an equivalent Au/Ti bowtie antenna with the same total length on a SiO_2_/HRSi substrate, where the SiO_2_ has a thickness of 300 nm, and HRSi has a thickness of 200 μm. The inset represents the central feeding area, where a thin (1 nm) HfO_2_ layer (with area of 300 × 300 nm^2^) is considered to form an MIM diode. The computed values of graphene’s complex surface impedance in the 400–1100 GHz frequency range are displayed in Figure 29c.

**Figure 30:**
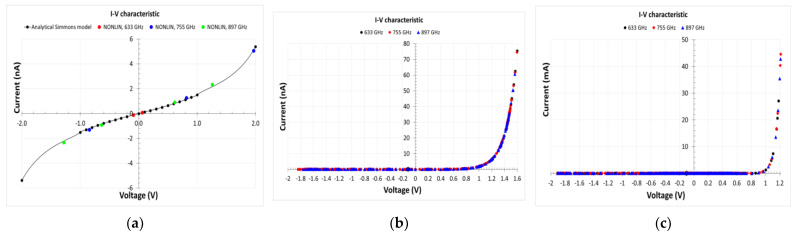
Current–voltage dependences of MIM and graphene/semiconductor diodes. (**a**) HfO_2_ MIM diode (the comparison with the analytical Simmons model is shown); (**b**) graphene/n-Si Schottky diode; (**c**) graphene/n-GaAs Schottky diode. Reproduced with permission from [22].

In Figure 30, we present the simulated current–voltage dependence of the 1 nm thick HfO_2_-based MIM diode (Figure 30a, along with a comparison with the analytical Simmons model), graphene/*n*-Si Schottky diode (Figure 30b), and graphene/*n*-GaAs Schottky diode (Figure 30c).

**Figure 31:**
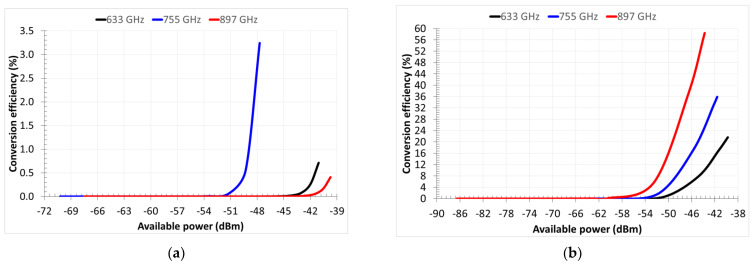
Simulated conversion efficiency for the (**a**) *n*-Si case and (**b**) *n*-GaAs case, at three frequencies: 633.8 GHz (solid black curve), 754.9 GHz (solid blue curve), and 897.1 GHz (solid red curve). Reproduced with permission from [22].

In Figure 31, we present the simulated conversion efficiency *η_THz-DC_* as a function of the available power *P_av_*. If *P_DC_* is the rectified power, *η_THz-DC_* is defined as *η_THz-DC_* = *P_DC_*/*P_av_*. The maximum value of *η_THz-DC_* is 3.24% for *P_av_* = −47.72 dBm at 754.9 GHz for the *n*-Si case, and *η_THz-DC_* = 58.43% for *P_av_* = −43.74 dBm at 897 GHz for the *n*-GaAs case. Thus, the advantage in using *n*-GaAs as the doped substrate is evident, due to the strong nonlinearity in the current–voltage characteristic. The conversion efficiency of the system with the MIM diode is not displayed because, in this case, it is always *η_THz-DC_* ˂˂ 1%.

## 3. Perspective and Conclusions

The entire manuscript demonstrates that nanomaterials such as CNTs, graphene, and MoS_2_ have enriched the research in the domain of efficient energy harvesters of EM waves from microwaves up to optical frequencies. RF energy harvesters exhibit a high conversion efficiency, exceeding 80% in the best cases. However, the most important sources of EM energy are in the infrared and visible spectrum ranges. Here, ultrafast devices are required to harvest the IR spectrum of the sun or the thermal radiation which is wasted from various sources. These devices are scarce, and impedance mismatch problems cause a strong degradation of the overall conversion efficiency. To solve this conundrum, we proposed a graphene self-rectifier antenna that, at THz frequencies, has a radiation efficiency of 1%, while the same antenna made of Au/Ti has a radiation efficiency of 95%. However, the self-rectifying graphene bowtie antenna on *n*-GaAs shows a conversion efficiency of 58.43% at 897 GHz, while the Au/Ti MIM-based rectenna (the metallic bowtie antenna with the same dimensions as the graphene one) has an efficiency much lower than 1%. It is now clear that new physical concepts must be found for EM energy harvesting in (and beyond) the THz frequency range, by fully exploiting the unique physical properties of existing and new atomically thin materials.

The harvesting of electromagnetic energy is dominated at microwaves and millimeter waves by complex circuits consisting of metallic antennas of different shapes, metallic antenna arrays, and semiconductor diodes, such as Schottky diodes and MIM diodes, attaining conversion efficiencies around 80–90%. These circuits can be used, for example, in Internet of things (IoT)-related applications requiring a multitude of tiny and low-power devices, which can be biased from rectennas replacing the batteries [66].

On the other hand, metallic antennas and metallic antenna arrays are being replaced by metasurface arrays [67] and antennas based on metamaterials [68] in order to increase the compactness and the efficiency of harvesters. Table 2 in [69] is a comprehensive synopsis of conversion efficiencies of various microwave and millimeter wave harvesters. The conversion efficiency depends on parameters such as the input power, frequency, and the gain of antennas, varying from 50% to 90%.

As we pointed out in our paper, the major ambient electromagnetic energy sources are the Sun and human-activity-related wasted heat and energy (see [28]); nevertheless, a huge amount of available energy located in the infrared spectrum is not collected by solar cells or by other harvesters. This is the main target for further research using rectennas in a world where an energy crisis is evidenced.

## Figures and Tables

**Table 1:** Physical properties of graphene [31].

Parameter	Value at Room Temperature	Applications
Mean free path	400 nm if the graphene is transferred on SiO_2_; higher than 1 μm or greater for graphene transferred on h-BN	Ballistic devices: harvesting microwaves, millimeter waves, and THz and IR energy
Mobility	40,000 cm^2^/V·s (intrinsic) 100,000 cm^2^/V·s in suspended graphene or in graphene deposited on h-BN Typical value: 8000 cm^2^/V·s	Transistors, high-frequency applications
Thermal conductivity	5000 W/m∙K, higher than in many metals	Thermal harvesting
Young’s modulus	1.5 TPa, 10× times greater than in steel	Stiff materials

**Table 2:** Characteristics of different growth methods for graphene [31].

Material	Method	Yield	Graphene Surface
Graphite	Repetitive peeling HOPG	Low	Small
SiC	Desorption of Si atoms at high temperature	Moderate	Moderate (3–4 inch wafers)
GO	GO dispersion into hydrazine	High	Large
CVD	Gas mixture (CH_4_ and H_2_)	Very high	Very large (30 inches)

**Table 3:** EM energy source for harvesting [51].

Material	Method	Yield
RF, microwaves, millimeter waves	100 μm–30 cm	2 μW/m^2^–10 mW/m^2^
Infrared thermal radiation	8–13 μm	0.1–1 mW/cm^2^
Sun	0.15—4 μm	5–100 mW/cm^2^

## Data Availability

The data presented in this study are available on request from the corresponding author.
